# Empathy Levels in Medical Students: A Single Center Study

**DOI:** 10.7759/cureus.38487

**Published:** 2023-05-03

**Authors:** Khalid Saifullah Baig, Muhammad Khizar Hayat, Mohammad Ahmed Arsalan Khan, Umer Humayun, Zunnoor Ahmad, Muhammad Afaq Khan

**Affiliations:** 1 Internal Medicine, Hayatabad Medical Complex, Peshawar, PAK; 2 Pediatric Surgery, Children's Health Ireland, Dublin, IRL; 3 Surgery, Tipperary University Hospital, Clonmel, IRL; 4 Anesthesia, Burns and Plastic Surgery Center, Peshawar, PAK; 5 General Surgery, Khyber Teaching Hospital, Peshawar, PAK; 6 Orthopaedics, Khyber Teaching Hospital, Peshawar, PAK

**Keywords:** teq score, empathy, undergraduate, medical, education

## Abstract

Objectives

To determine the level of empathy in medical students and to determine the difference in empathy levels between the two genders in a single center.

Materials & methods

This qualitative study was conducted at a medical college in Peshawar from March 2021 to July 2021. Institutional ethical committee approval was taken (RMI/RMI-REC/Approval/83) before commencing the study. All students admitted into the medical college in the current academic year 2020 to 2021 were included in the study. Any students that did not fill out the questionnaire completely were excluded. The Toronto Empathy Questionnaire (TEQ) was used in this study. The questionnaire was uploaded on google forms for data collection. All the resulting scores were entered into IBM SPSS version 23.0. The mean TEQ score was calculated. Box and whisker plots were made for respective years. An Independent sample t-test was used to determine the association between mean TEQ scores and gender.

Results

Of 367 students, 347 (94.6%) participated in this study, with a slight female predominance (53%). The mean age of the students was 21.44 (SD = 1.751) years. The participation rate was *≥*70% from each class. Most participants across the years have an above-average empathy score (49.9%). Among the participants, the year I (67.6%) showed most participants with high empathy. Year IV (40.6%) has the highest proportion of below-average empathy scores. The mean empathy score of female students was 49.08 (S.D = 7.588), while the empathy score for male students was 44.59 (S.D = 7.58).

Conclusion

Empathy levels decline as medical education is progressed through the years. Females show a greater sense of empathy than their male counterparts. A slight increase in empathy levels is seen in the final year of medical school after a decline over the initial years.

## Introduction

The patient-doctor relationship is the cornerstone for the provision of efficient and quality health care. Empathy is one of the must-have competencies in a doctor, and communication is one of the modalities that plays a vital role in achieving it [1].

Empathy, in medicine, is defined as a physician's understanding of a patient's experience and his/her ability to converse the feeling back [2]. A clinical relational connection must be established to obtain optimum clinical results [3]. It is shown in the literature that empathy involves the domains of placing yourself in the patients' shoes, perspective-taking, and compassionate care. Empathic engagement can be achieved in patient care if these domains are followed [4].

The need to assess empathy and develop it in medical students and physicians was backed up by Neumann et al., who discovered that physicians' empathy had a positive preventative effect on depression in cancer patients [5]. Sir William Osler also iterated that the physician requires a clear mind and an affectionate heart; his work is difficult and intricate, requiring the use of the highest cognitive abilities while constantly appealing to emotions and finer feelings [6].

Studies on emotional intelligence and empathy conducted on medical students have shown a decline in their level as the years progress [7]. One of the studies concluded that empathy in post-clinical years is lower than that in pre-clinical years [8]. A few suggestions as to why this was as follows, demanding work environment, emotional exhaustion, lack of self-awareness or belief, or the lack of knowledge of the importance of empathy [9].

The declining empathy trend among medical students and physicians is a growing concern for medical educationists worldwide, and this has been backed up by many studies [10,11]. As empathy is measurable and teachable, a challenge lies ahead for medical educationists to devise a strategy to overcome this task [12]. One strategy is to teach students about ethics, humanity, and professionalism, which was demonstrated in one study to significantly positively affect their future working practices [13]. Another strategy is integrating empathy and assessing it within the training program [4]. Workshops on communication skills and patient-doctor relationship is another strategy that can be adopted and have been shown to positively influence the participants [13].

Empathy development is a slow process and develops over time. This process starts with listening, reasoning, and understanding, followed by communication of the awareness empathically and, finally, the perception of being understood by the counterpart [14]. Pakistan's medical education system mainly focuses on providing the required knowledge to pursue medicine but pays little attention to humanistic skills [5]. This was proven by a study done in Lahore which showed that the current medical curriculum of Pakistan lacks the domain of development of empathy [15]. This study was conducted to determine the level of empathy in medical students throughout their medical curriculum and to determine differences in empathy levels between males and females.

## Materials and methods

This descriptive cross-sectional study was conducted at a medical college in Peshawar from March 2021 to July 2021. Institutional ethical committee approval was taken (RMI/RMI-REC/Approval/83) before commencing the study. All students admitted into the medical college in the current academic year 2020 to 2021 were included in the study. Any student that did not fill out the questionnaire completely was excluded. World Health Organization sample size calculator was used for calculating the sample [16]. A sample size of 500 was used with a confidence interval of 95%. The minimum sample size required was 217.

The Toronto Empathy Questionnaire (TEQ) was used in this study. The questionnaire was uploaded on google forms for data collection. The link generated from the online form was shared with the students on their official college email addresses with the help of the Student Affairs section of the college. Individual students from each year were also contacted to share the questionnaire link in their respective class groups on social media. The questionnaire was left open for one month to provide adequate time.

Data collected was downloaded from Google Forms in Microsoft Excel format and converted to IBM SPSS format. Any duplicate entries that were noted were removed from the data file. A total for each questionnaire was obtained. Items 1, 3, 5, 6, 8, 9, 13, and 16 were scored on a 5-point scale for positively worded responses. Always = 4; Frequently = 3; Occasionally = 2; Rarely = 1; Never = 0. The following items with negative wording were reverse scored: 2, 4, 7, 10, 11, 12, 14, 15. The Toronto Empathy Questionnaire (TEQ) total was calculated by adding the scores ranging from 0 to 64. The average score of males for this criterion ranges from 43.46 to 44.45, while females' average score varies from 44.62 to 48.93. Gender disparities are reported to be moderate, according to this questionnaire.

All the resultant scores were entered into IBM SPSS version 23.0. The mean TEQ score was calculated, and box and whisker plots were made for respective years. An Independent sample t-test was utilized to assess the statistical significance of the association between genders and mean Toronto empathy scores. A multi-regression analysis was also conducted.

## Results

Out of 367 responses, 347 (94.6%) responses from the students were included in this study as per the inclusion and exclusion criteria. A slight female predominance (53%) was appreciated in response. The mean age of the students was 21.44 years, with a standard deviation of 1.751. The participation rate was greater than 70% from each class. All participants from Years I and II consented to the study, while fewer students consented to be part of the study from Year IV (84.3%). Table 1 shows the demographics.

**Table 1:** Demographics (Only Consented Participants) *Participation rate of ≥70% from each class

		N (%)
Age (Years)		21.44 ± 1.75
Gender	Males	164 (47.3)
	Females	183 (52.7)
Response from Year of Education (Total Participants)*	Year I (74)	74 (100)
	Year II (70)	70 (100)
	Year III (72)	70 (97.2)
	Year IV (70)	59 (84.3)
	Final Year (81)	74 (91.3)

Table 2 shows the empathy scores of all participants in the study. Most participants had an above-average empathy score (49.9%) across the years. Among the participants, year I (67.6%) showed most participants with high empathy, and year IV (40.6%) had the highest proportion of below-average empathy scores. A trend of decreasing empathy scores can be seen in the above-average section, while the opposite is seen in below average section barring the final year.

**Table 2:** Empathy Scores per Year

Year of Education	Below Average (110) n (%)	Average (64) n (%)	Above Average (173) n (%)
Year I (74)	14 (18.9)	10 (13.5)	50 (67.6)
Year II (70)	23 (32.9)	11 (15.7)	36 (51.4)
Year III (70)	27 (38.6)	9 (12.9)	34 (48.6)
Year IV (59)	24 (40.6)	14 (23.7)	21 (35.6)
Final Year (74)	22 (29.7)	20 (27.0)	32 (43.2)

**Figure 1:**
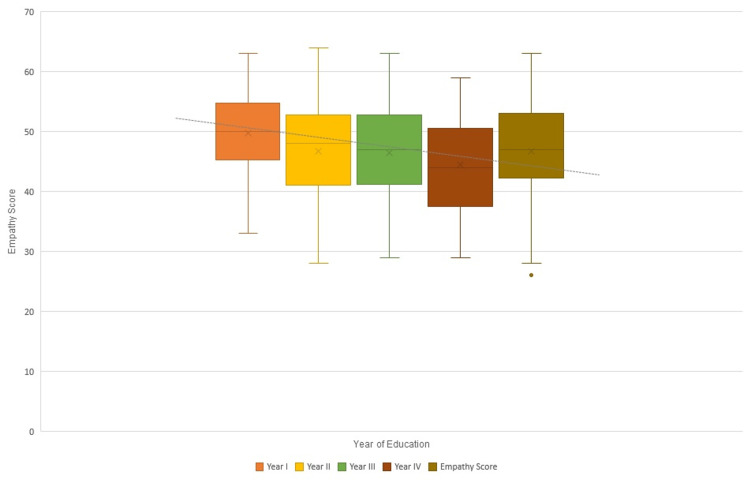
Empathy Score and Year of Education (correlation coefficient = –1.36)

Figure 1 shows a box and whisker plot of the mean empathy scores against the year of education. A slight decrease in the mean empathy scores can be seen from the best-fit line. The correlation coefficient is -1.36 (statistically significant at p<0.05).

Table 3 shows that the mean empathy score of female students was 49.08 (S.D = 7.588), while the empathy score for male students was 44.59 (S.D = 7.58). It shows that in terms of comparison according to gender, the empathy score was higher among female students than male students. The difference between the male and female students was found to be statistically significant (p<0.001).

**Table 3:** Empathy Score Comparison on Gender (p<0.001)

Gender	Mean	Standard Deviation	Standard Error	95% Confidence Interval for mean	
				Upper Bound	Lower Bound
Male	44.59	8.203	0.641	43.32	45.85
Female	49.08	7.588	0.561	47.98	50.19

Multiple linear regression was run to predict empathy scores based on gender, age, year of education (MBBS), and marital status. The multiple regression model statistically significantly predicted empathy scores, i.e., F(4, 342) = 9.654, p < .0005, adj. R2 = .09. Moreover, the results of the multiple regression analysis suggest that among all the four variables, only gender significantly predicts empathy scores, i.e., p<0.0005. The slope coefficient (B) is -4.69, which means predicted empathy scores for males are 4.69 units less than their female counterparts. The results of the multiple linear regression are given in Table 4.

**Table 4:** Multiple regression results for Empathy Scores

		95% CI for B				Sig.		
	B	LL	UL	SE B	β		R^2^	Adjusted R^2^
Model							0.10	0.09
(Constant)	47.97	29.98	65.96	9.15		0.000		
Gender	-4.69	-6.38	-3.00	0.86	-0.29	0.000		
Age	0.01	-0.87	0.89	0.45	0.00	0.981		
Year of Education (MBBS)	-0.90	-1.97	0.17	0.54	-0.16	0.099		
Marital Status	3.61	-4.14	11.35	3.94	0.05	0.360		

## Discussion

Empathy in the patient-doctor connection is linked to many favorable outcomes for the doctor and the patient. Approaching patients empathically is stressed in medical faculties' education curricula. The relevance of developing empathy levels in medical students is demonstrated by clinical empathy, which is a crucial component of patient-doctor communication, improves patient satisfaction, reduces anxiety, and improves clinical outcomes [1].

A cross-sectional empathy profile of medical students is presented in this study during their 5-years at a medical college in Peshawar, Pakistan. Our sample included about an equal male-female ratio, 1:1.1. The mean empathy score in our study was found to be 46.96±8.188 which was less than that found by Akgün, Ö et al. inTurkey, i.e., 52.8 ± 6.1 [1]. Another study done on Indian medical students showed a mean TEQ score of 39.28 ± 15.65, which is significantly lower than our scores [17]. A study conducted by Riaz S et al. conducted. In Lahore, Pakistan showed the mean empathy scores of the students, which were less than those observed in our study [5]. Two other surveys in Pakistan showed mean TEQ scores of 39.61 ± 6.54 and 44.24 ± 6.59 [11].

Our study also showed that the empathy pattern decreases as we progress through the years in medical college until the fourth year, after which it shows a slight improvement in the final year of medical college. This could be due to the increased patient-student interaction during their ward rounds. The drop in empathy levels can be due to the continued loss of idealism, excessive workload, and a scarcity of good role models [1]. However, there is an improvement in the empathy levels in the final year from the fourth year; this is still less than that of students in the first year (49.76). Similar results were also demonstrated in the studies conducted by Youssef FF et al. In the Caribbean in 2014 and by Khademalhosseini et al. from Iran in 2014 [18,19]. Youssef et al., Haque et al., and Stefanovic et al., also report similar findings through the TEQ empathy scores. A significant decrease in empathy levels as the education process progressed from the first to the final year of medical school [6,20]. Whereases Akgün Ö et al. found a rise in empathy scores from 1st year to 4th year and then a dip during 5th and 6th year of medical school, contrary to most studies [1]. It should, however, be noted that the duration and curricula are different in the Akgün Ö et al. study. Riaz S et al. study demonstrated that the mean TEQ score kept declining throughout the 5 years of MBBS except in the fourth professional year of MBBS [5].

Our study found that 4th-year medical students had the lowest score in the TEQ scoring system with a mean of 44.53±8.26. This was also the case in another study conducted in Malaysia by Haque M et al. [6]. Youssef FF et al. and Akgün Ö et al. found dissimilar results with 6th and 5th-year students, respectively, in their studies had the least TEQ score [1,18].

Many studies found that female students' empathy scores were higher than male fellows [1,5,6,18-22]. Our study also illustrated that the females have higher empathy scores on TEQ than males (44.59 ± 8.20) and females (49.08 ± 7.59).

Using the TEQ and other tools, Bhatia G et al. in India and Imran SS et al. in Pakistan also concluded that empathy scores according to TEQ decline as the year progresses [17,23]. The low degree of empathy among medical students and its decline as their medical education advances has been a source of worry in several research. Studies on how to raise empathy levels in medical students have been done because of this concern. Empathy can be increased by successful educational interventions, targeted training programs, or medical interviews, according to studies [1].

Limitations

This was a single-center study, and the results can only be generalized to this center. Similar studies should be conducted on a large scale to determine a causal relationship. The patients' demographics in this center may have influenced the empathy score, and a more varied representation of the sociodemographic background of students should be included in the study. A snapshot in time has been taken about the data available; following the students in their first year till their final year will make for a better study. Only some demographic factors were considered; a more extensive study must be conducted to evaluate other humanistic and environmental factors influencing empathy.

Recommendation

These findings indicate that our medical curriculum and educational strategies should be reviewed, lead efforts, and spend more time on psychophysiological education to prevent the loss of empathy and promote empathy during medical school. In Pakistan, medical educators should search for ways to incorporate emotional intelligence into the curriculum, which will help to promote patient-centered care, patient satisfaction, and good communication skills. Real-life community exposure is critical to enhancing empathy in undergraduate medical students. Other relevant psychophysiological and behavioral techniques should be incorporated to measure empathy development. These might enable us to understand better the cognitive and emotional changes that students go through during and after their training.

Assessment of pre and post-intern year/house job empathy level assessment should be done to assess if increased interaction with patients in a work environment increases empathy levels among fresh graduates.

## Conclusions

Empathy levels decline as medical education is progressed through the years. Females show a greater sense of empathy than their male counterparts. A slight increase in empathy levels is seen in the final year of medical school after a decline over the initial years.
